# Sensory acuity and cross-language phonetic similarity jointly predict second language vowel production accuracy

**DOI:** 10.3389/fnhum.2026.1744572

**Published:** 2026-04-22

**Authors:** Joanne Jingwen Li, Tara McAllister, Douglas M. Shiller, Xing Tian, Maria I. Grigos

**Affiliations:** 1 Department of Communicative Disorders and Sciences, San José State University, San Jose, CA, United States; 2 Department of Communicative Sciences and Disorders, New York University, New York, NY, United States; 3 School of Speech-Language Pathology and Audiology, Université de Montréal, Montréal, QC, Canada; 4 NYU-ECNU Institute of Brain and Cognitive Science at NYU Shanghai, Shanghai, China; 5 Division of Arts and Sciences, New York University Shanghai, Shanghai, China

**Keywords:** auditory acuity, L2 speech production, production variability, somatosensory acuity, speech motor control

## Abstract

**Introduction:**

Research on second language (L2) speech perception and production has yielded mixed findings, suggesting that factors beyond perceptual ability influence L2 production outcomes. This study investigated predictors of inter-speaker variability in L2 vowel production, focusing on individual differences in auditory and somatosensory acuity. The roles of phonological awareness and trial-to-trial production variability were also examined.

**Methods:**

Forty English-speaking adult late learners of Mandarin produced two Mandarin vowels: /u/ (phonetically similar to English /u/) and /y/ (a novel vowel for English speakers). Production accuracy and trial-to-trial variability were measured acoustically. Auditory acuity was assessed using speech identification and discrimination tasks. Somatosensory acuity was measured through a Phonetic Awareness Task (PAT) and a novel Tongue Placement Task (TPT). Linear mixed-effects models were used to identify predictors of production accuracy.

**Results:**

Predictors of production accuracy differed by vowel. For the perceptually similar vowel /u/, lower production variability was the only significant predictor of higher accuracy. For the novel vowel /y/, higher somatosensory acuity (PAT) was the only significant predictor of accuracy. No predictors significantly accounted for production variability in either vowel.

**Discussion:**

These findings suggest that sensory acuity and L1–L2 phonetic similarity jointly constrain L2 speech learning. Specifically, somatosensory acuity supports the establishment of accurate articulatory targets for novel L2 sounds, while production variability reflects the stability of learners’ phonetic category formation for perceptually similar L2 sounds.

## 1 Introduction

Many adult L2 learners do not achieve native-like pronunciation of L2 sounds, which is often attributed to difficulties in the perception of L2 sounds (Aoyama et al., 2004; Hao and de Jong, 2016; Jia et al., 2006). Even experienced L2 learners tend to assimilate L2 sounds to their perceptually similar native categories and approximate L2 sounds to their L1 counterparts in production (Flege, 1995, 1996, 2002). However, many studies have reported a weak relationship between L2 perception and production (e.g., Kartushina and Frauenfelder, 2014; Peperkamp and Bouchon, 2011; Sakai and Moorman, 2018; Saito and van Poeteren, 2018). Given these mixed results, the present work aimed to investigate factors that may influence a speaker’s success in L2 production. Primarily, we examined the effect of auditory acuity, somatosensory acuity, production variability, and phonological awareness on L2 production in late L2 learners. The effect of perceptual similarity between L2 targets and L1 vowels was also explored.

### 1.1 Effects of auditory and somatosensory acuity on L1 production

The current investigation was guided by the theoretical framework of the Directions Into Velocities of Articulators (DIVA) model, which proposes that speech sounds have both auditory goals, associated with acoustic signals of sounds (e.g., formant frequencies), and somatosensory goals that indicate the place and degree of constriction formed by articulators within the vocal tract (Guenther, 1994, 1995; Guenther et al., 1998, 2006; Guenther, 2016). Auditory and somatosensory goals are target regions with an acceptable range of variability in the respective sensory space, and the narrowness of the sensory target regions is associated with phonetic precision in the production of the sounds (Guenther, 1994, 1995; Guenther et al., 1998, 2006; Guenther, 2016).

From a neurobiological perspective, based on DIVA, auditory and somatosensory goals are sensory target regions within distributed cortical networks that support speech perception and motor control (Guenther, 2016; Tourville and Guenther, 2011). Auditory goals have been associated with posterior temporal regions involved in acoustic–phonetic processing, whereas somatosensory goals are supported by somatosensory and parietal regions involved in articulatory state estimation. Recent physiological and neuroanatomical evidence suggests that this somatosensory involvement is supported by an extensive neural infrastructure of auditory-somatosensory interactions that exists across the entire auditory hierarchy (Franken et al., 2022). Within this framework, individual differences in sensory acuity may influence the precision with which these sensory targets are specified, such that higher acuity is associated with more tightly defined target regions and increased sensitivity to sensory feedback. Furthermore, because speech sounds can be decoded directly from activity in the somatosensory cortex, somatic units may play a functional role in mapping acoustic patterns onto phonetic categories (Franken et al., 2022). More precise sensory targets may, in turn, support more effective use of feedback-based mechanisms to guide speech motor control.

Consistent with this theoretical framework, some empirical studies have shown that speakers’ varying auditory and/or somatosensory acuity are related to individual differences in speech production for both vowels (Franken et al., 2017; Perkell et al., 2004a, 2008; Villacorta et al., 2007) and consonants (Ghosh et al., 2010; Newman et al., 2001; Perkell et al., 2004b). For example, speakers with higher auditory acuity were found to produce native vowel contrasts with longer between-phoneme acoustic distance and smaller within-phoneme variability (Franken et al., 2017; Perkell et al., 2008). Furthermore, it was found that speakers who are better at perceptually discriminating the sibilant contrast /s/ and /ʃ/ produced the sounds with greater acoustic and articulatory distinction (Ghosh et al., 2010; Perkell et al., 2004b). A possible interpretation of these findings is that speakers with higher sensory acuity are more likely to detect deviations from the prototype in their sensory feedback and make corrections, resulting in a narrower sensory target region and more consistent sound production. This is consistent with Villacorta et al. (2007), who reported that speakers showing more acute auditory discrimination of vowels differing in F1 frequency adapted more to F1-altered auditory feedback in production. Research has also suggested that auditory and somatosensory acuity are independent of one another (Nasir and Ostry, 2008) and that speakers vary in the degree of reliance on each of the sensory domains for speech production (Lametti et al., 2012). Therefore, in the current study we hypothesized that individual differences in auditory and somatosensory acuity may also explain inter-speaker variability in L2 speech production.

### 1.2 Effects of auditory and somatosensory acuity on L2 production

The effect of auditory acuity on L2 production is related to the widely examined L2 perception-production link. As mentioned earlier, research findings in this area are mixed. Many studies found that L2 learners who show poor auditory discrimination of L2 contrasts often have difficulty with the production of those sounds (Aoyama et al., 2004; Hao and de Jong, 2016; Kartushina et al., 2015; Jia et al., 2006; Sebastián-Gallés and Baus, 2017; Wang et al., 2003). On the other hand, a dissociation between L2 perception and production has been widely reported as well (Golestani and Pallier, 2007; Kartushina and Frauenfelder, 2014; Li et al., 2019; Lopez-Soto and Kewley-Port, 2009; Peperkamp and Bouchon, 2011). Even in studies that reported an L2 perception-production connection, the cross-domain correlation at an individual level was often weak (e.g., Kartushina and Frauenfelder, 2014; Kartushina et al., 2015). It is worth noting that somatosensory acuity was not systematically investigated in most of the above-mentioned studies, and it is possible that the disassociation between L2 auditory perception and production is due to an influence from a speakers’ somatosensory skills.

To date, Li et al. (2019) remains the only study to have simultaneously examined auditory and somatosensory acuity in relation to nonnative speech sound learning. In that study, English-speaking participants were trained to produce two Mandarin vowels (/y/ and /u/) using either visual–acoustic biofeedback or ultrasound biofeedback. Visual–acoustic biofeedback provides learners with a real-time display of their own speech acoustics (e.g., vowel formant frequencies) alongside target values, whereas ultrasound biofeedback provides real-time visual information about tongue movement, with a target tongue configuration superimposed for learners to match.

Li et al. hypothesized that learners with lower auditory acuity would show greater improvement in production accuracy when trained with visual–acoustic biofeedback, which primarily provides information in the auditory–acoustic domain. Conversely, they expected that learners with lower somatosensory acuity would benefit more from ultrasound biofeedback, which emphasizes articulatory information. However, the results revealed no significant associations between sensory acuity and gains in production accuracy. Furthermore, there were no significant interactions between sensory acuity and biofeedback type. These null findings may reflect, at least in part, limitations in the measurement approaches used to assess auditory and somatosensory acuity.

In that study, auditory acuity was assessed using a just-noticeable-difference (JND) discrimination task involving stimuli positioned near the /y/ end of a synthesized Mandarin /y/−/u/ continuum. Because Mandarin /u/ proved more challenging for English learners, the use of stimuli clustered around /y/ may not have sufficiently taxed perceptual sensitivity. Consistent with this interpretation, participants exhibited a ceiling effect in their auditory JND scores. Similarly, somatosensory acuity was evaluated using a stereognosis task, which primarily indexes tactile discrimination relevant to consonantal articulation rather than vowel production.

To address these methodological limitations, the present study employs more rigorous and context-appropriate assessments, including a more demanding auditory discrimination task to evaluate auditory acuity and proprioception-based measures to assess somatosensory acuity. The use of proprioception-based measures aligns with established psychophysical research on jaw position discrimination and interdental dimension awareness, which indicates that the somatosensory system possesses high-resolution acuity for detecting subtle changes in mandibular and lingual positioning (Hannam and Tobias, 1980; Kent, 2024). Such measures may more accurately capture the sensory precision required for vowel height and backness contrasts compared to traditional tactile stereognosis tasks.

### 1.3 Effects of characteristics of target sounds on L2 production

A large body of research in L2 speech learning has emphasized that the perceptual and articulatory properties of target sounds play an important role in shaping learning outcomes. According to the Speech Learning Model (SLM; Flege, 1995), L1 and L2 sounds are represented within a shared phonological space, and successful L2 acquisition requires learners to maintain contrastive distinctions between categories across languages. Similarly, the Perceptual Assimilation Model (PAM; Best, 1994) proposes that non-native speech sounds are perceived relative to existing L1 phonological categories and are assimilated based on their perceived articulatory and acoustic similarity. Extending this framework to L2 learners (PAM-L2), Best and Tyler (2007) suggest that initial perceptual assimilation patterns may persist with experience and influence subsequent L2 speech learning. In both frameworks, when an L2 sound closely resembles an existing L1 category (i.e., a similar sound), learners are likely to assimilate it to the nearest L1 category rather than treat it as phonologically distinct, which can result in reduced perceptual sensitivity and persistent difficulty in accurate L2 production. In contrast, L2 sounds without a close L1 counterpart (i.e., a new sound), are often more amenable to learning over time, as learners can establish new phonetic categories for these sounds.

In line with these theoretical accounts, empirical findings also suggest that the predictors of L2 speech learning may vary depending on the phonetic status of the target sound. For example, Li et al. (2019) observed that while auditory and somatosensory acuity did not significantly predict learners’ production gains, post-training trial-to-trial production variability and phonological awareness were significantly associated with improvements in production accuracy. Critically, these effects differed between the Mandarin vowels /u/ and /y/, with /y/ lacking a perceptually similar English counterpart. This pattern reinforces the idea that the identity of the target sound, specifically, whether it is perceptually similar to or distinct from an L1 category, can modulate the factors that contribute to successful L2 production.

Motivated by this theoretical and empirical foundation, the present study examines how perceptual similarity between L2 vowels and L1 categories influences the role of learner-specific factors in L2 vowel production.

### 1.4 Effect of phonological awareness on L2 production

Phonological awareness refers to language learners’ awareness of and the ability to analyze speech units, which has been suggested to be positively associated with accuracy in speech perception (Perrachione et al., 2011) and production (Hu, 2003; Hu et al., 2013; Li et al., 2019; Mora et al., 2014).

Such association may be explained by Schmidt’s Noticing Hypothesis (Schmidt, 1990, 1992), which suggests that awareness of a second language progresses along a continuum, ranging from unconscious perception to conscious noticing and explicit understanding. Mora et al. (2014) suggest that to successfully acquire L2 phonology, one must be able to perceive the acoustic contrasts between L1 and L2 sounds and consciously notice these distinctions, thereby transforming perceptual sensitivity into the ability to imitate. In their study, Spanish learners of English were asked to mimic an English accent when producing Spanish words with initial voiceless stops (/p/, /t/, & /k/). They also produced English words and Spanish words with the same initial stops. The results showed that the voiceless stops in both English words and English-accented Spanish words had longer voice onset times (VOTs) than those in Spanish words. This was interpreted as evidence that the speakers had developed a “noticing” level of phonological awareness. The authors argued that this awareness enabled speakers to notice the acoustic differences between English and Spanish VOTs and to imitate English pronunciation accordingly.

Similarly, Li et al. (2019) reported that phonological awareness was a significant predictor of progress in production accuracy for Mandarin /u/ but not /y/. They suggested that since Mandarin /u/ is perceptually similar to English /u/, a higher level of phonological awareness was necessary for English speakers to notice and adjust for the subtle difference between the two vowel categories. Taken together, these findings suggest that phonological awareness plays an important role in L2 speech production. Accordingly, this study explored phonological awareness as a potential factor influencing L2 vowel production accuracy.

### 1.5 The role of production variability in speech learning

In addition to production accuracy, increasing attention has been paid to production variability as a theoretically meaningful individual-difference measure in speech learning. Production variability refers to token-to-token dispersion in repeated realizations of the same speech sound and has been argued to reflect the stability or compactness of a speaker’s phonetic representations rather than random noise (Kartushina and Frauenfelder, 2014; Kartushina et al., 2016).

In the context of L2 speech learning, several studies have demonstrated a relationship between production variability and acquisition outcomes, suggesting that variability captures important aspects of speech motor control and category formation (Kartushina and Frauenfelder, 2014; Kartushina et al., 2016; Li et al., 2019). Kartushina and Frauenfelder (2014) reported that speakers with lower variability in L1 vowel production produced L2 vowels more accurately, and Kartushina et al. (2016) further showed that lower variability was associated with improved learning of non-native vowel contrasts. More recently, Li et al. (2019) found that lower production variability was predictive of greater improvement in L2 vowel production following biofeedback-based training, supporting the view that variability indexes a stable individual characteristic relevant to speech learning rather than task-specific error.

Importantly, production variability is related to, but not solely explained by, sensory acuity. Although production variability has been shown to correlate with perceptual discrimination in some contexts (e.g., Perkell et al., 2004a, 2004b; Franken et al., 2017), it is also influenced by articulatory stability and motor control (Kartushina and Frauenfelder, 2014). Within sensorimotor models of speech production, such as the DIVA framework (Guenther, 1994, 1995, 2016), speech targets are represented as regions in sensory space; speakers with narrower target regions are predicted to exhibit both lower production variability and greater production accuracy (Perkell et al., 2004a, 2004b; Guenther et al., 2006). Consequently, production variability provides a complementary window into individual differences in speech learning that is distinct from explicit measures of auditory or somatosensory acuity.

From a neural perspective, production variability can be interpreted as reflecting the stability of sensorimotor representations that support speech execution. Feedforward motor commands are refined through practice and become increasingly stable, reducing reliance on sensory feedback for online correction (Guenther, 2016; Tourville and Guenther, 2011). Thus, lower trial-to-trial production variability may index more stable feedforward representations that support consistent and accurate speech motor execution. Crucially, the development and refinement of these feedforward representations depend on sensory feedback during learning, such that auditory and somatosensory acuity constrain the quality of error signals available for calibrating motor commands. Individuals with greater sensory acuity may therefore develop more precise sensorimotor mappings, resulting in both reduced production variability and greater production accuracy. Conversely, increased variability may reflect less stable sensorimotor integration or greater reliance on feedback-based control, which can limit production accuracy, particularly for non-native speech sounds that require the establishment of new articulatory patterns. Although the present study does not directly assess neural activity, this framework provides a principled account of how individual differences in production variability, sensory acuity, and production accuracy may be linked through shared neural mechanisms supporting speech motor control.

Given this theoretical and empirical background, the present study treats production variability as a key construct. Specifically, variability is examined both as a predictor of L2 vowel production accuracy and as an outcome measure, allowing us to assess how individual differences in variability relate to accuracy across different vowel types and whether variability itself is systematically associated with other learner characteristics.

### 1.6 The current study

The current study had two primary aims. The first was to investigate predictors of inter-speaker variability in L2 vowel production accuracy in late L2 learners. Guided by the DIVA framework (Guenther, 1994, 1995, 2016), we examined speakers’ auditory and somatosensory acuity as the main hypothesized predictors of L2 vowel production accuracy. The effects of phonological awareness and L2 vowel production variability were also examined since both have been suggested to play important roles in L2 production (Li et al., 2019; Kartushina and Frauenfelder, 2014). The second aim was to understand the role that the characteristics of target sounds play in L2 speech sound learning. Predictors of L2 vowel production accuracy for new vs. similar L2 vowels were examined separately.

We predicted that for new L2 vowels, speakers with higher sensory acuity would be more likely to establish accurate sensory targets for the targets, and as a result, both auditory and somatosensory acuity would be significant predictors of L2 vowel production accuracy. This prediction aligns with SLM, which posits that speakers need to learn the phonetic features of and establish new categories for new L2 vowels, and the DIVA model, which argues that speech sounds have both auditory and somatosensory targets. We also predicted that L2 production accuracy would be negatively correlated with L2 vowel production variability (Kartushina and Frauenfelder, 2014; Li et al., 2019). The effect of phonological awareness was not expected to have a significant effect on production accuracy because strong phonological awareness skills should not be required for L2 learners to notice the phonetic differences between the L2 targets (new L2 vowels) and L1 categories.

For perceptually similar L2 vowels, we predicted that speakers might produce the vowels with their L1 counterparts, so individual speakers’ L2 vowel production accuracy (distance from L2 production to target distribution) was not expected to vary with their sensory acuity or production variability. Additionally, phonological awareness was expected to be a significant predictor of production accuracy because participants with strong phonological awareness skills are more likely to “notice” the perceived subtle phonetic difference between L2 and L1 vowels as compared to individuals with weaker phonological awareness skills.

Finally, given the theoretical importance of production variability as a potential index of phonetic stability, the present study included an exploratory analysis of variability as an outcome measure. It remains an open question whether production variability represents a relatively stable, trait-like characteristic of an individual’s motor system or a state-like feature that is directly shaped by sensory acuity and linguistic knowledge. While sensorimotor models such as DIVA suggest that higher sensory acuity should lead to more stable motor execution through refined feedback-to-feedforward mapping, this relationship has not been consistently demonstrated in experienced L2 learners. Therefore, we explored whether individual differences in sensory acuity, phonological awareness, and L2 learning experience were systematically associated with L2 vowel production variability. This analysis was intended to clarify the nature of motor stability in late L2 learners and determine the extent to which it remains linked to the other learner characteristics assessed in this study.

## 2 Methods

### 2.1 Participants

Forty English native speakers, between 18–40 years of age (31 females, 9 males), participated in the study. The participants were late learners of Mandarin, who did not learn or receive regular exposure to Mandarin before middle school (around 12 years of age). Their Mandarin learning experience ranged from 4 months to 12 years (mean = 4.37 yr., SD = 2.79 yr) and self-reported Mandarin proficiency ranged from 1 to 6 (on a 7-point Likert scale) (mean = 3, SD = 1.40). The participants had no regular exposure to languages that involve a front-back contrast in high rounded vowels that are similar to the Mandarin vowels (/y/ vs. /u/) examined in the current study. All participants reported having no phonetic training and no history of neurobehavioral, speech, hearing, and language disorders. Eighteen Mandarin native speakers between 18–40 years of age (9 males, 9 females) served as control speakers. The Mandarin speakers were matched with the English speakers based on their height because vowel formants are correlated with vocal tract length, which is suggested to be associated with a speakers’ height (Fitch, 1997; Fitch and Giedd, 1999). Each gender group included three tall speakers (>170 cm for females; >180 cm for males), three medium height speakers (160-170 cm for females, 170-180 cm for males), and three short speakers (<160 cm for females; <170 cm for males). All participants signed an informed consent form before participating in the experiments. This study was approved (IRB-FY2020-4250) by the Institutional Review Board at New York University.

### 2.2 Stimuli

This work examined the production of Mandarin high back rounded vowel /u/ and high front rounded vowel /y/ by English late learners of Mandarin. Mandarin /u/ is phonetically similar to English /u/, while Mandarin /y/ is a new vowel category for English speakers (Chang et al., 2011; Xie and Jaeger, 2020). Therefore, examining the production of the Mandarin /u/ and /y/ by English speakers allowed us to investigate whether predictors of L2 vowel production accuracy differ for L2 vowels that do and do not have similar L1 sounds.

A reading task was used to elicit the English and Mandarin speakers’ productions of Mandarin /u/ and /y/, which were embedded in monosyllabic words *wù 物* ([u^51^] “thing”) (51refers to the Mandarin high-falling tone) and *yù遇* ([y^51^], “meet”), respectively. The words were presented in both Pinyin and the corresponding Chinese characters. Frequently used Chinese characters were chosen for the phonological shapes of the items so that the English speakers would be familiar with the stimuli. In addition, among the four lexical tones in Mandarin, the high-falling tone was selected for the items because it is similar to the falling pitch contour in English monosyllabic words (Chang et al., 2011).

### 2.3 Procedures

Participants completed an online session conducted through Zoom, followed by an in-person session at the Department of Communicative Sciences and Disorders at New York University.

#### 2.3.1 Online session

During the online session, participants completed a questionnaire (available at: https://osf.io/gy8ez/) that included questions about their language learning experience, such as age at which they started to learn Mandarin, frequency of Mandarin use outside classroom, and self-reported proficiency in Mandarin. Afterwards, participants’ phonological awareness was assessed using three subtests from the *Comprehensive Test of Phonological Processing—Second Edition (CTOPP-2)* (Wagner et al., 2013): Elision, Blending, and Phoneme Isolation. The elision task required removing one or more segments from a word and forming a new word (e.g., *say “toothbrush” without saying “tooth”*). The blending task involved combining segments or syllables together to form new words (e.g., *What word do these sounds make? Cow-boy*). In the phoneme isolation task, participants were asked to identify a phoneme at certain position in a word (e.g., *What is the first sound in the word “man”?*).

#### 2.3.2 In-person session

During the in-person session, participants were first administered a pure-tone hearing screening (20 dB at 500 Hz, 1,000 Hz, 2000 Hz, and 4,000 Hz, for both ears). Participants then completed a word reading task designed to elicit productions of the target sounds, along with tasks assessing auditory and somatosensory acuity.

##### 2.3.2.1 Word reading task

The word reading task was administered through Gorilla Experiment Builder^1^ (Anwyl-Irvine et al., 2021). During this task, participants were instructed to read aloud words presented on a computer screen. There were 20 blocks of four English words (total of 80 trials), followed by 20 blocks of six Mandarin words (total of 120 trials) that included the two targets (*wù 物* & *yù遇*) for the present study. Trials within each block were played in a randomized order. Participants wore a closed-back over-the-ear Sennheiser headphone (HMD 280 PRO) with an integrated microphone that was adjusted to be 2-inches to the mouth. The headset was connected to a MacBook Pro (version 11.6) via a USB-C audio interface (MOTU M2), and the productions were recorded into Praat (Boersma and Weenink, 2016) using a 44.1 kHz sampling rate.

##### 2.3.2.2 Auditory acuity measures

Following the word reading task, participants completed a combination of a speech identification task and an AXB staircase discrimination task in MATLAB (The Mathworks, Natick, MA) to assess their auditory acuity. Stimuli for the two tasks were sounds from 480 equally spaced tokens on a synthesized continuum between the Mandarin /u/ and /y/ vowel categories. The continuum was generated by adjusting all formants between two natural productions of Mandarin /u/ and /y/ from a female Mandarin speaker, using the speech algorithm STRAIGHT (Speech Transformation and Representation by Adaptive Interpolation of weiGHTed spectrogram) (Kawahara et al., 2013). The speech identification task was conducted first to identify each participant’s perceptual boundary between Mandarin /u/ and /y/. During the task, participants heard sounds that were selected from 10 equally spaced intervals between two ends on the 480-step Mandarin /u/−/y/ continuum. Each sound was repeated ten times, for a total of 100 trials, and all sounds were presented in a randomized order. Upon completing the task, each participant received an immediately generated value representing the step number of the stimuli that received 50% response rate for /y/, and this value was considered the participant’s perceptual boundary between Mandarin /u/ and /y/ vowel categories.

Following the identification task, participants completed the AXB staircase discrimination task, in which they discriminated stimuli around their own perceptual boundary on the /u/−/y/ synthesized continuum. During the task, participants heard the sound sequence A-X-B in each trial and were asked to identify which of A and B was different from X. X was the sound at each participant’s perceptual boundary for Mandarin /u/−/y/. Either A or B was identical to X, while the other was different and selected from either side (/u/ or /y/) of the boundary on the continuum. The task followed an adaptive staircase procedure (Ghosh et al., 2010; Schouten et al., 2003; Villacorta et al., 2007): the between-stimuli distance began with 80 steps and went down after every three consecutive correct responses and up after every incorrect response. The size of the stimulus change was eight steps down and four steps up for the first four reversals, four-down and two-up for the second four reversals, and two-down and one-up for the third four reversals. The task ended after 12 reversals or a maximum of 80 trials.

##### 2.3.2.3 Somatosensory acuity measures

Somatosensory acuity was assessed through two tasks: a phonetic awareness task (PAT) (McAllister et al., 2020; Gritsyk et al., 2021) and a novel tongue placement task (TPT). PAT, run in PsychoPy3 (v13; Peirce et al., 2019), required participants to judge tongue positions for English sounds across four blocks of nine trials. In Block 1, they identified whether a consonant (e.g., “z” in “zero”) was produced with the front or back of the tongue. Each trial included orthographic and audio instructions, audio models, and three repetitions before making a keyboard response. In the remaining blocks, participants compared two vowels (e.g., “ey” in “hate” vs. “oh” in “hoed”), indicating which was further back (Block 2), higher (Block 3), or lower (Block 4), after repeating them alternately three times. If a target sound was mispronounced on the first attempt, the experimenter corrected it, and the participant repeated it once before continuing.

TPT, the second task for measuring somatosensory acuity, was conducted through Gorilla Experiment Builder. Participants produced a sound while their auditory feedback was masked, which required them to rely on their proprioceptive awareness to reach the target position. The masking noise was speech-shaped with an intensity level of 80–85 dB (Niziolek et al., 2015; Parrell et al., 2021). The noise was presented through closed-back, over-the-ear headphones that were connected to a MacBook Pro via the audio interface. To ensure that the noise effectively masked the participants’ speech, a volume testing session was conducted prior to the somatosensory acuity task. During this session, participants were asked to read the Rainbow Passage aloud using a comfortable but quiet (non-whispered) volume at which they could not hear themselves. They received visual feedback via a volume unit meter displaying their sound level in dB SPL and were instructed to maintain this volume throughout the subsequent task.

**Figure 1:**
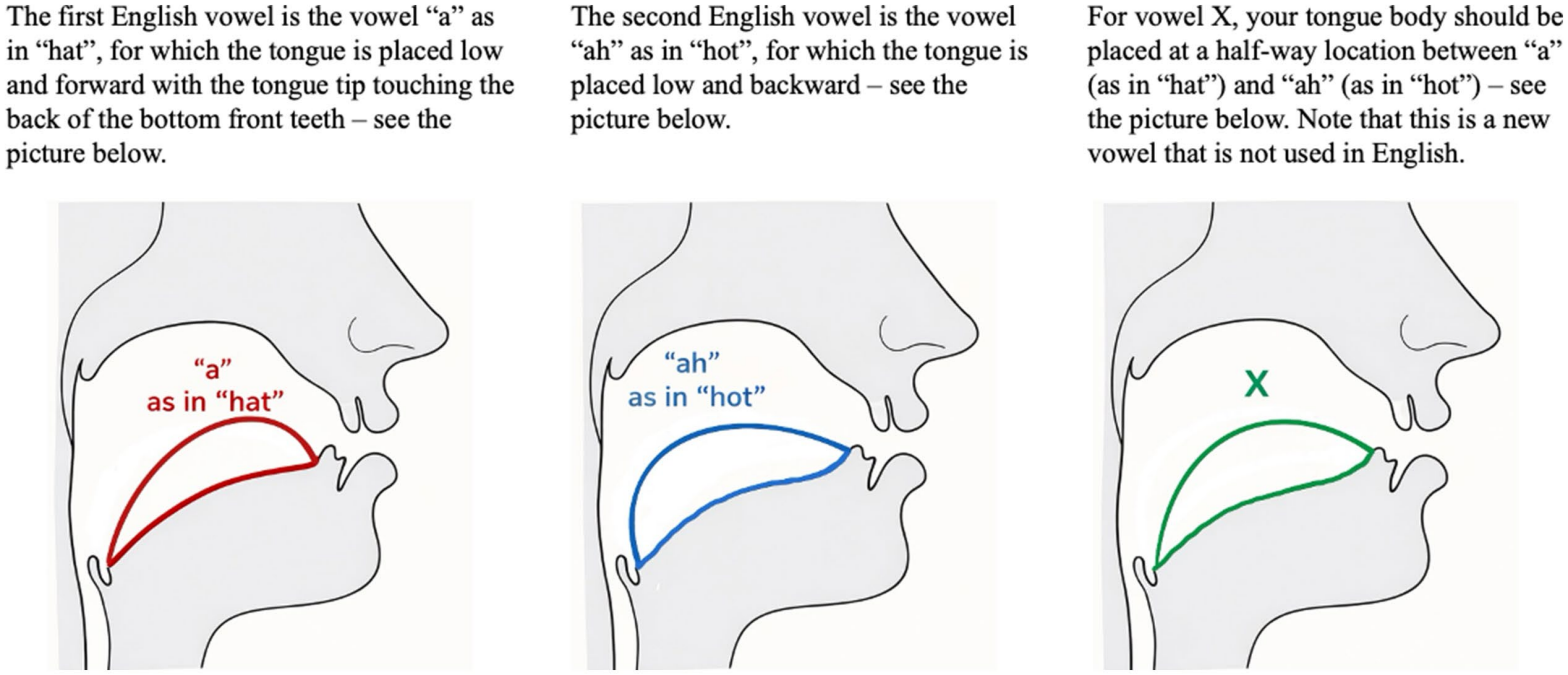
Schematic diagrams of tongue placement for English /æ/, /ɑ/, and the novel vowel X, with accompanying participant instructions. Illustrations created by the author based on standard phonetic descriptions (see Ladefoged, 2006).

After volume testing, participants were instructed to produce the English vowels /æ/ and /ɑ/, which contrast in tongue position (front vs. back), followed by a novel vowel X, with the tongue placed midway between /æ/ and /ɑ/. Orthographic descriptions and vocal tract diagrams (Figure 1) illustrated the target tongue placements.

Participants first practiced the vowels silently, then produced 20 repetitions of /æ/−/ɑ/−X or /ɑ/−/æ/−X sequences under masked auditory feedback. The /æ/−/ɑ/ order was counterbalanced within subjects, and trials were randomized.

### 2.4 Measurement

#### 2.4.1 L2 vowel production accuracy and variability

The acoustic analyses of the Mandarin vowels /u/ and /y/ were conducted using Praat (Boersma and Weenink, 2016). The onset and offset were automatically identified for each vowel using the intensity-based annotation function in Praat with a silence threshold of -25 dB. The experimenter visually inspected the following: (1) the onset and offset of each vowel, which was manually corrected, if needed; (2) the alignment between Praat’s formant tracking and the energy concentration on the spectrogram, and a linear predictive coding (LPC) filter order associated with the best alignment was selected for each speaker and vowel. The first two formant frequencies (F1 & F2) were extracted at the vowel midpoint using a Praat script (Lennes, 2003) with the selected filter order. Outliers were visually inspected and manually corrected if they deviated from expected ranges. Final F1 and F2 values were used to compute production accuracy and variability, analyzed in R (R Core Team, 2020).

The F1 and F2 values in Hertz were transformed into the psychoacoustic Bark scale using the “vowels” package (Kendall and Thomas, 2018). The center of the distributions of productions for each vowel was calculated for the Mandarin speakers in each height group, which was used as the target for the height-matched English speakers. For each English speaker and vowel, the Euclidean Distance (ED) in F1-F2 space from each production to the target was measured. ED values that fell at least 2.5 median absolute deviations above or below the median ED (Leys et al., 2013) within each individual speaker and vowel were considered outliers and discarded. A total of 21 data points (1.3%) were identified as outliers and eliminated. A smaller ED value indicates that a vowel production is closer to the target distribution, indicating a higher accuracy, and vice versa. Therefore, vowel production accuracy is represented as ED in the following analysis.

English speakers’ token-to-token production variability of each target vowel was also measured, which was the area of the elliptical distribution of the vowel repetitions in F1-F2 space. The area was calculated as a 95% confidence interval around the multivariate mean of the distribution. A smaller area indicates a lower variability, and vice versa. Vowel production variability is represented as Variability in the following analysis.

#### 2.4.2 Auditory acuity

Each participant’s auditory acuity was represented as the just noticeable distance (JND), the average between-stimuli distance across the last 4 reversals in the AXB staircase discrimination task. A lower JND score indicates higher auditory acuity.

#### 2.4.3 Somatosensory acuity

##### 2.4.3.1 PAT

The percentage of correctness across all trials in PAT represents each participant’s somatosensory acuity score. A higher PAT score indicates higher somatosensory acuity.

##### 2.4.3.2 TPT

Two somatosensory acuity measures were derived from TPT: variability (TPT-Variability) and accuracy (TPT-ED). With auditory feedback masked, cross-trial production variability of the novel vowel X was hypothesized to reflect proprioceptive awareness. Specifically, speakers with greater awareness of tongue position relative to the oral cavity should be able to reproduce that position more consistently. To assess this, we calculated the token-to-token production variability (TPT-Variability) of the novel vowel X—measured as the area of the elliptical distribution of the 20 repetitions in F1-F2 space. A smaller area (i.e., lower variability) indicates higher somatosensory acuity.

The second measure, TPT-ED, assessed production accuracy relative to a predefined articulatory target. For each participant, we first identified the median F1-F2 values for their productions of English /æ/ and /ɑ/ across 20 repetitions each. The midpoint between these two medians served as the target position for vowel X. We then calculated the Euclidean distance (ED) from each token of vowel X to this midpoint and took the median of these distances as the TPT-ED score. A smaller TPT-ED score reflects greater accuracy in hitting the intended tongue position and, thus, higher somatosensory acuity.

#### 2.4.4 Phonological awareness

Phonological awareness was represented by the Phonological Awareness Composite Score (PACS) derived from the three subtests (Elision, Blending and Phoneme Isolation) of CTOPP-2 (Wagner et al., 2013). A higher PACS is associated with stronger phonological awareness skills.

### 2.5 Statistical analysis

The purpose of the analysis was to examine predictors of L2 vowel production accuracy. Complete data can be found at: https://osf.io/9qt5j/. Descriptive statistics were calculated for all sensory acuity, phonological awareness, and production variability measures. A Wilcoxon signed-rank test was used to compare production variability between the two Mandarin vowels. To compare production accuracy across vowels, a linear mixed-effects model was fitted using the lmer() function in the lmerTest package (Kuznetsova et al., 2017), with Euclidean distance (ED; reverse of production accuracy) as the dependent variable, vowel as a fixed effect, and random intercepts and slopes for vowel by subject.

To examine predictors of L2 vowel production accuracy, separate linear mixed-effects models were fitted for each Mandarin vowel (/u/ and /y/), given the *a priori* hypothesis that predictors would differ for perceptually similar versus new L2 vowels. In each model, ED relative to the target distribution was the dependent variable. Fixed effects included years of Mandarin study (L2_exposure) and self-reported Mandarin proficiency (Proficiency) to control for individual differences in L2 learning experience, as well as auditory acuity (JND), somatosensory acuity (TPT-Variability, TPT-ED, and PAT), phonological awareness (PACS), and vowel production variability. A random intercept for subject was included to account for repeated observations within speakers. All models converged successfully using the default optimizer, with no warnings or indications of singular fit. Multicollinearity was assessed using variance inflation factors (VIFs) from the car package (Daoud, 2017), and all VIF values were below 2.

In addition, linear regression models were conducted to examine predictors of vowel production variability for each Mandarin vowel separately. In these models, production variability served as the dependent variable, and predictors included years of Mandarin study, self-reported proficiency, auditory acuity, somatosensory acuity measures, and phonological awareness. Multicollinearity was again assessed using VIFs and found to be low across all predictors.

## 3 Results

### 3.1 Descriptive statistics

The mean, standard deviation, and range (min, max) are reported in Table 1 for each sensory acuity, phonological awareness, and production variability measure, which indicate that each measure was reasonably dispersed across participants. The Shapiro–Wilk tests for normality showed that some variables, particularly TPT-Variability and the variability measures of /y/, were not normally distributed, suggesting a possible ceiling performance.

**Table 1:** Descriptive statistics of sensory acuity, phonological awareness, and production variability measures.

Measure	Mean	SD	Min	Max	Shapiro–Wilk *p*-value
Auditory (just noticeable difference)	22.68	11.03	5	49.75	0.43
Somatosensory (TPT_variability)	3.72	3	0.28	12.43	<0.001
Somatosensory (TPT_ED)	1.36	0.54	0.40	3.01	0.03
Somatosensory (PAT)	72.50	11.57	41.18	94.12	<0.01
Phonological awareness (PACS)	101.38	11.07	77	122	0.47
Variability_u (area of the ellipse)	2.02	1.06	0.36	4.27	0.12
Variability_y (area of the ellipse)	1.64	2.29	0.22	14.96	<0.001

**Figure 2:**
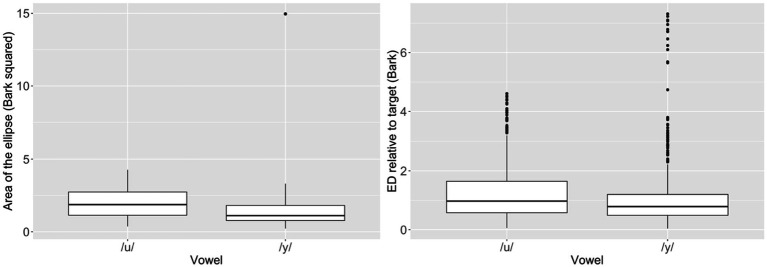
Area of the ellipse (production variability) (left) and ED relative to target (reverse of accuracy) (right), separated by vowel.

The Wilcoxon signed-rank test showed that the production variability of /y/ was significantly lower than that of /u/ (*V* = 593, *p* = 0.01) (see left panel in Figure 2). The linear mixed-effects model did not show significant difference in ED (reverse of production accuracy) between the two vowels (*β* = −0.23, SE = 0.17, *p* = 0.19) (see right panel in Figure 2).

### 3.2 Predictors of L2 production accuracy

Linear mixed-effects models were used to examine predictors of L2 vowel production accuracy (ED from the target distribution). VIF values were below 2 for all predictors, suggesting low multicollinearity risk in these models (Daoud, 2017). All mixed-effects models converged successfully using the default optimizer, with no warnings or indications of singular fit.

**Figure 3:**
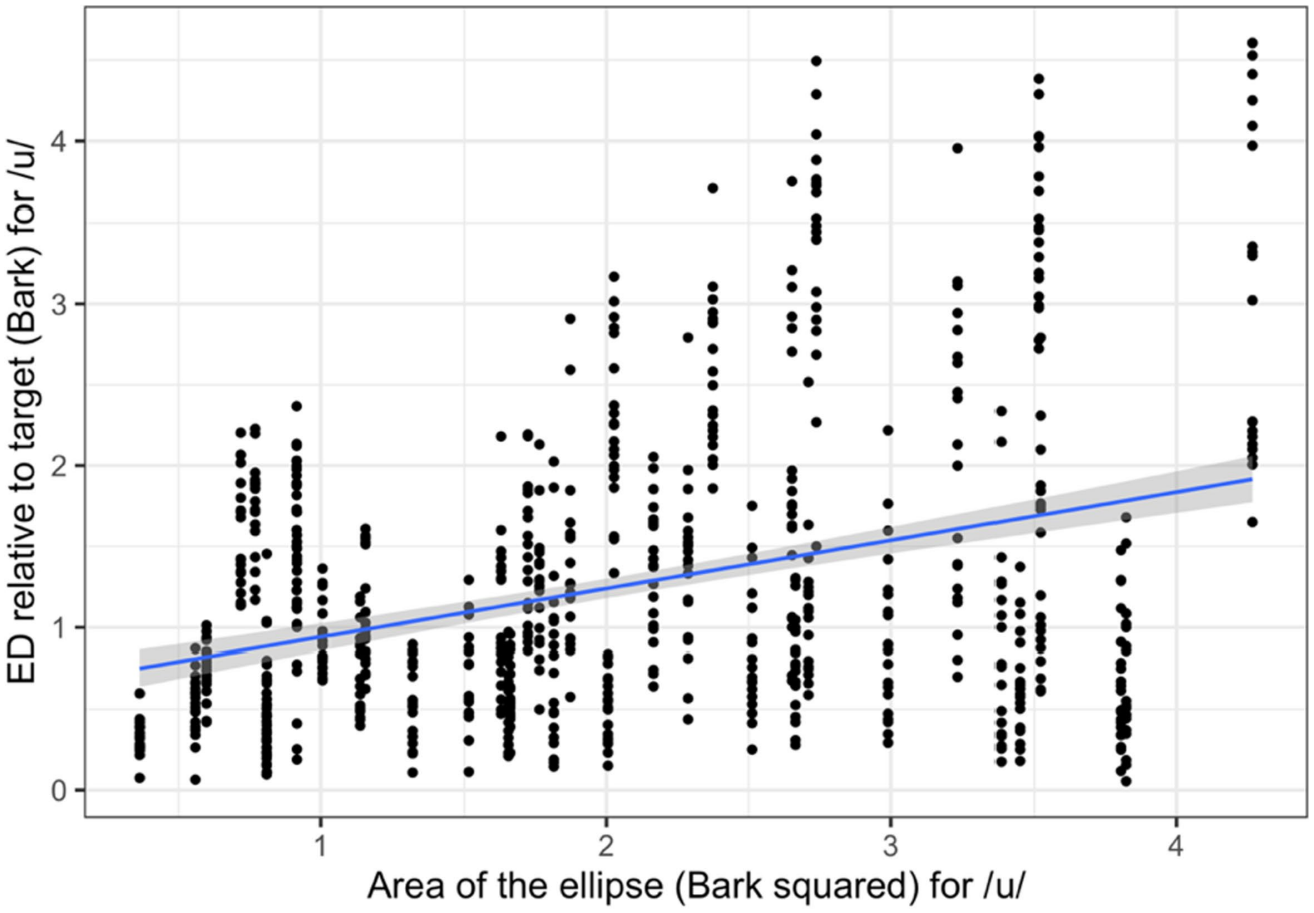
Association between area of the ellipse (production variability) and ED relative to target (reverse of production accuracy) for Mandarin /u/.

For the Mandarin vowel /u/, Variability was the only significant effect (*β* = 0.31, SE = 0.13, *p* = 0.02). The coefficient was positive, indicating that speakers who produced Mandarin vowels with lower variability tended to have higher production accuracy (lower ED values) (see Figure 3). None of the other predictors were significant.

**Figure 4:**
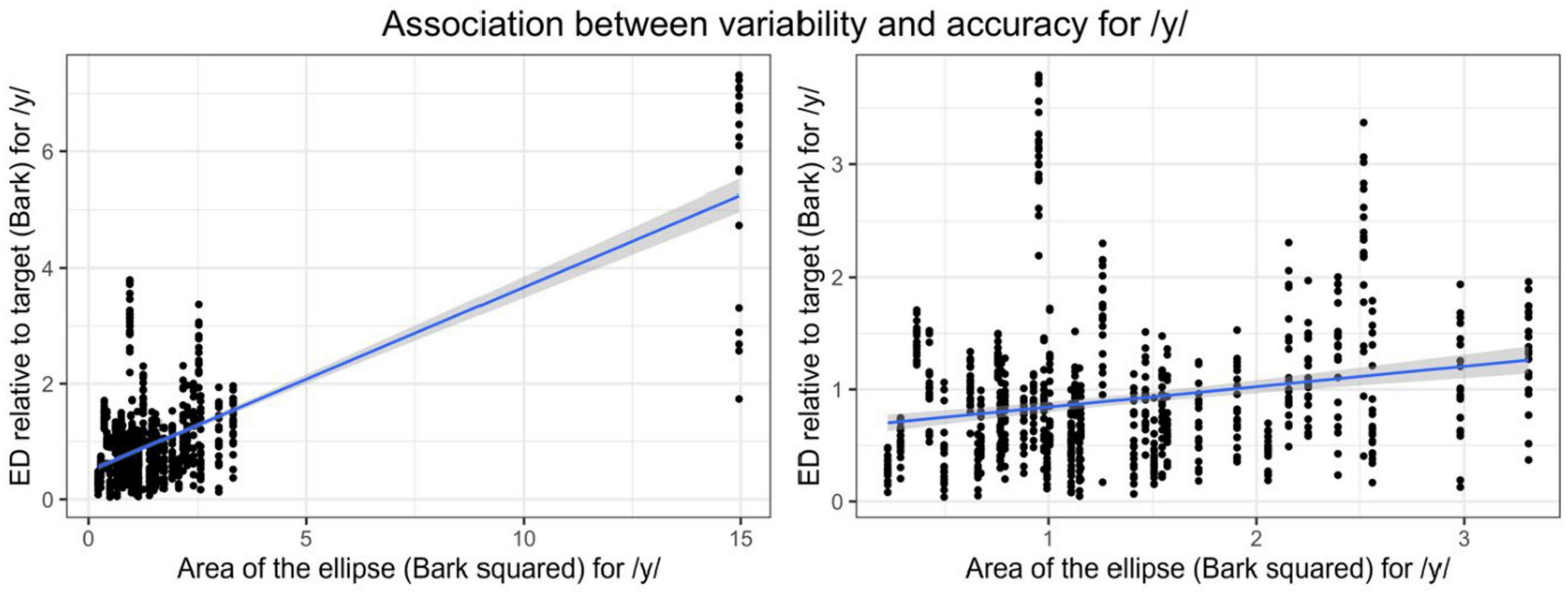
Association between area of the ellipse (production variability) and ED relative to target (reverse of production accuracy) for Mandarin /y/ with participant 1025 included (left) and excluded (right) respectively.

**Figure 5:**
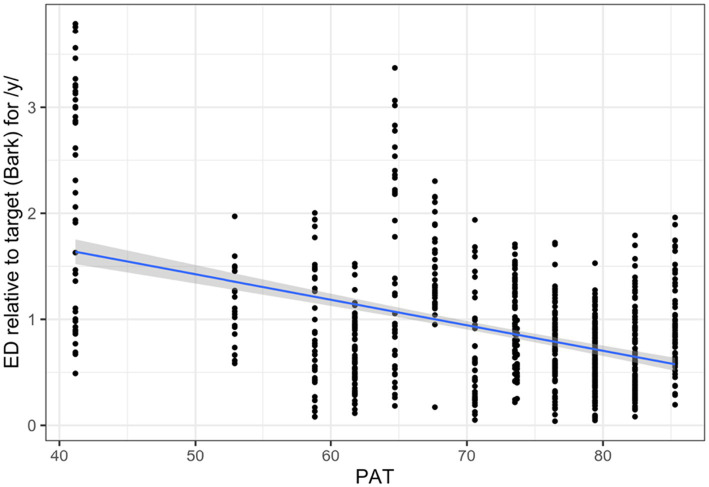
Association between somatosensory acuity measured by PAT and ED relative to target (reverse of production accuracy) for Mandarin /y/ with participant 1025 excluded.

For Mandarin /y/, both PAT (*β* = −0.02, SE = 0.01, *p* = 0.02) and Variability (*β* = 0.36, SE = 0.04, *p* < 0.001) were significant. However, this Variability effect appeared driven by an influential data point from participant 1025 (Figure 4, left). After excluding this participant, Variability was no longer significant (Figure 4, right), and PAT was the only significant predictor (*β* = −0.02, SE = 0.01, *p* = 0.01), indicating that higher PAT scores predicted better production accuracy (lower ED; Figure 5).

### 3.3 Predictors of L2 production variability

Linear regression models were used to examine predictors of production variability for Mandarin /u/ and /y/ separately. In the analysis for /y/, participant 1025 was excluded. Multicollinearity was low in both models, with variance inflation factor (VIF) values for all predictors below 2.

For both Mandarin /u/ and /y/, none of the examined predictors reached statistical significance, indicating that production variability was not significantly associated with participants’ L2 learning experience, auditory acuity, somatosensory acuity, or phonological awareness.

## 4 Discussion

This study investigated individual differences in L2 vowel production accuracy, focusing on how sensory acuity, phonological awareness, and production variability relate to production outcomes. Guided by the DIVA model (Guenther, 1994, 1995, 2016) and L2 learning models (e.g., SLM, Flege, 1995; PAM-L2, Best and Tyler, 2007), we examined English-speaking learners’ production of two Mandarin vowels: /u/, which is perceptually similar to English /u/, and /y/, which lacks an English counterpart. We hypothesized that the predictors of L2 production accuracy would differ depending on the perceptual similarity between L1 and L2 vowels. Specifically, we expected that for Mandarin /u/, phonological awareness would play a stronger role than sensory acuity, reflecting learners’ reliance on L1-based categories, whereas for Mandarin /y/, higher auditory and somatosensory acuity and lower production variability would be associated with more accurate productions. Overall, the results partially supported these hypotheses, revealing distinct patterns of predictors for the two vowels consistent with their degree of L1–L2 similarity.

### 4.1 Production variability correlates with accuracy for /u/ but not /y/

Contrary to our prediction, production variability emerged as a significant predictor of production accuracy for Mandarin /u/: speakers who produced more consistent (i.e., less variable) vowels also had smaller acoustic distances from native targets. Our findings suggest that many experienced learners did not assimilate Mandarin /u/ to English /u/ but instead treated it as a distinct category. This interpretation is supported by the descriptive data, which showed greater variability for /u/ than for the perceptually new vowel /y/, as well as a roughly normal distribution of variability values for /u/, rather than a distribution skewed toward uniformly low variability. Although Mandarin /u/ is perceptually similar to English /u/, the observed relationship between higher accuracy and lower variability remains consistent with the SLM and PAM-L2, which allows for the formation of new L2 categories when learners can perceive sufficient phonetic distinctions between L2 sounds and their L1 counterparts (Flege, 2002). In this context, lower variability may reflect more stable phonetic representations for learners who are further along in establishing a distinct Mandarin /u/ category within a shared phonological space.

These results partially align with those of Kartushina and Frauenfelder (2014), who reported a significant association between variability and accuracy for one of two perceptually similar French vowels among Spanish speakers with several years of French learning experience. This similarity may reflect that learners with more L2 experience begin to form new phonetic categories for perceptually similar vowels, during which increasing accuracy is often accompanied by decreasing variability.

By contrast, the current findings differ from those of Li et al. (2019), who found no correlation between variability and accuracy change among Mandarin-naïve English learners after short-term training on Mandarin /u/. One possible reason for this difference may be the participants’ level of experience. In Li et al. (2019), the beginner learners likely relied on L1-based categories, leading to stronger L2-to-L1 assimilation. In contrast, more experienced learners, as in Kartushina and Frauenfelder (2014) and the present study, may have developed new L2 categories for perceptually similar vowels, resulting in a closer relationship between production accuracy and variability.

For Mandarin /y/, production variability was not a significant predictor of production accuracy, which was also unexpected. The null result may be due to a ceiling effect in variability, as evidenced by a skewed distribution (Table 1). When an outlier (participant 1025) who had both the highest variability and the least Mandarin learning experience (four months) was included, a significant relationship emerged, suggesting that speakers’ L2 learning experience plays a role in stabilizing category formation. This interpretation is consistent with Li et al. (2019), where Mandarin-naïve English learners showed a strong association between variability and accuracy for /y/, likely because they were still in the early stages of forming a new phonetic category. Taken together, the results suggest that most participants in the current study had already established a relatively stable phonetic category for /y/ and were producing it with consistency.

Overall, our results suggest that for experienced learners, the vowel /y/ had mostly stabilized, whereas /u/, which is perceptually close to English /u/, still showed considerable individual variation. Learners who produced /u/ more accurately and consistently may be further along in developing a separate phonetic category for it. This pattern is consistent with SLM (Flege, 1995, 1996, 2002), which proposes that L2 sounds that are perceptually similar to L1 sounds (like /u/) are harder to acquire than sounds that are perceptually new (like /y/).

### 4.2 Phonological awareness does not predict accuracy in experienced learners

Phonological awareness did not significantly predict production accuracy for either vowel. This was expected for /y/ but not /u/, for which we had anticipated a positive relationship based on Schmidt’s Noticing Hypothesis (Schmidt, 1990, 1992) and previous findings (e.g., Mora et al., 2014; Li et al., 2019). According to Schmidt, phonological awareness encompasses multiple levels, from the unconscious level of “perceiving” (e.g., detecting cross-language acoustic differences), to “noticing” (being aware of what has been perceived), and finally to “understanding” (the ability to analyze and articulate the observed differences). We hypothesized that English speakers with higher phonological awareness would be more likely to notice the acoustic differences between Mandarin /u/ and English /u/, thus facilitating the formation of a new phonetic category.

The absence of this effect in our data may reflect the advanced L2 learning status of our participants, who had likely already internalized the distinction between L1 and L2 vowels through learning experience such as classroom instruction (Kissling, 2013; Saito, 2011; Shively, 2008; Spada and Tomita, 2010). In contrast, in Li et al. (2019), participants had no prior Mandarin learning experience, and phonological awareness played a stronger role. This pattern is consistent with the Perceptual Assimilation Model-L2 (Best and Tyler, 2007), which suggests that experienced L2 learners, unlike naïve learners, may possess abstract phonological knowledge (e.g., L1/L2 phonemic inventory and orthographic system) from various sources to guide their perception of L1-L2 similarity (also see Chang, 2019). Taken together, our findings suggest that phonological awareness may play a critical role in learning perceptually similar L2 vowels during the early stages of acquisition, but its influence diminishes as learners gain more experience.

### 4.3 Auditory acuity shows no relationship with production accuracy

Auditory acuity did not significantly predict production accuracy for either vowel, which was unexpected. We hypothesized a positive association between auditory acuity and production accuracy for /y/, drawing from prior research suggesting links between L2 perception and production (Flege, 1995, 1996, 2002; Ingram and Park, 1997; Aoyama et al., 2004), as well as from theoretical assumptions of the DIVA model (Guenther, 1994, 1995, 2016). According to DIVA, speakers with higher sensory acuity are expected to form narrower targets for speech sounds, which should lead to more accurate and precise speech productions.

A possible explanation for the null findings is a ceiling effect in the production accuracy for /y/, which was reflected in the highly skewed distribution in ED (relative to target) (right panel in Figure 2). Considering that all participants were experienced Mandarin learners and that isolated /y/ is relatively easy to produce accurately, it is possible that most speakers had already achieved near-native production for this vowel, leaving little room for individual differences in auditory acuity to manifest. Within the DIVA framework, auditory targets are thought to guide early stages of category formation, whereas somatosensory targets may play a more prominent role once auditory goals are established. At later stages of learning, variation in production accuracy may therefore be driven more strongly by somatosensory factors than by auditory perception.

We also believe that the null result was not due to the design of the auditory acuity measures, which were intentionally more demanding than those used in Li et al. (2019) and aimed to avoid ceiling effects in perceptual scores. In fact, Shapiro–Wilk tests confirmed that the JND scores generated in the auditory acuity tasks were normally distributed (Table 1), ruling out ceiling effects in the outcome measures. To better understand the perceptual contribution to production at various stages of L2 learning, future studies should compare learners with more strictly controlled L2 experience levels and incorporate a wider range of speech stimuli, potentially including both easy and difficult, as well as perceptually similar and new L2 sounds.

### 4.4 Somatosensory acuity predicts accuracy for /y/ in PAT but not in TPT

Of the three somatosensory acuity measures included simultaneously in the model, only PAT significantly predicted production accuracy for Mandarin /y/. This finding suggests that explicit articulatory awareness, as indexed by PAT, plays a particularly important role in producing perceptually new L2 vowels with high accuracy. This result is consistent with the DIVA framework, which emphasizes the role of somatosensory targets in guiding speech production once auditory goals are established.

The absence of significant effects for the two TPT-based measures was unexpected. One possible explanation is that this task may tap into a different dimension of somatosensory ability than PAT. Specifically, TPT may reflect a more implicit form of articulatory awareness or motor control skill, whereas PAT requires explicit, metalinguistic knowledge about tongue positions. This notion aligns with what Gritsyk et al. (2021) proposed regarding their bite block task, and it may also help explain why there was no strong correlation among the three somatosensory measures we used. Implicit articulatory awareness or motor control skills may be more relevant during earlier stages of L2 learning, when learners are still acquiring sensorimotor mappings for unfamiliar sounds and lack sufficient articulatory knowledge to verbalize or consciously evaluate their productions. Given that TPT is a relatively new measure, further research is needed to evaluate its sensitivity and validity as an index of somatosensory acuity for speech. Future studies combining acoustic analyses with articulatory tracking techniques, such as ultrasound imaging or electromagnetic articulography, may provide clearer insight into how different dimensions of somatosensory acuity relate to speech production accuracy.

### 4.5 Production variability shows no systematic association with sensory acuity or linguistic measures

Finally, when production variability itself was examined as an outcome measure, none of the sensory acuity, phonological awareness, or L2 learning experience variables emerged as significant predictors for either vowel. This pattern is consistent with a previous finding by Cheng et al. (2021), who similarly found no correlation between perceptual acuity and two measures of vowel production variability. Cheng et al. (2021) suggested that one possible explanation is that production variability may reflect individual differences in the stability of motor execution rather than in the precision of the underlying sensory target. That is, variability may be driven more by the consistency with which a speaker executes a given motor plan than by the size of the auditory category associated with that sound.

This interpretation does not, however, imply that production variability is uniform across all phonetic categories. Chao et al. (2019) demonstrated a strong relationship between vowel variability and the location of the category boundary in perceptual space. As they acknowledged, the direction of causality in that relationship is not clear. One possibility is that perception constrains production, such that vowels with less perceptual territory are produced with lower variability. Another possibility is that production shapes perception, and variability in production determines where the categorical boundary falls. If the latter is true, the question arises as to why some vowels are more variable than others, given that motor noise is a relatively stable individual-level trait. One possibility is that some vowels provide more somatosensory feedback than others. High front vowels, for example, are notable for a relatively well-defined somatosensory target (Perkell, 2012), which may constrain the range of allowable variability for those sounds.

Taken together, we suggest that each vowel has a maximum possible magnitude of variability, which is influenced by proximity to other vowels as well as by auditory and somatosensory feedback. Within that allowable range, the speaker’s relatively stable motor consistency determines where they fall. These two perspectives operate at different levels of analysis and are compatible with one another. This distinction is also consistent with our finding that variability predicted accuracy for /u/ but not for /y/. For /y/, most participants had already established a well-defined sensory category for this vowel, supported by both auditory and somatosensory targets, resulting in a ceiling effect in variability (skewed distribution; Table 1). Individual differences in motor consistency were therefore too small to predict accuracy. For /u/, where considerable individual variation remained, speakers who were more consistent in their productions also tended to be more accurate. This pattern reflects individual differences in how far along each learner is in establishing a stable sensory category for Mandarin /u/ that is distinct from the L1 /u/ category, rather than differences in the absolute level of variability across the two vowels. Taken together, these findings support the view that motor consistency is a relatively stable individual characteristic, while acknowledging that the degree to which it predicts accuracy depends on the stage of sensory category formation for a given vowel.

### 4.6 Neural interpretations of individual differences in L2 speech production

The observed associations between production accuracy, somatosensory acuity, and production variability may reflect individual differences in the precision and stability of sensorimotor representations supporting L2 speech. Neurobiological models of speech production propose that accurate speech relies on the integration of sensory feedback with motor commands within distributed cortical networks, with speech targets represented as regions in sensory space (Guenther, 1995, 2016; Tourville and Guenther, 2011). Within this framework, individuals with more precise somatosensory representations may specify narrower articulatory target regions, supporting greater production accuracy. This interpretation is consistent with our finding that somatosensory acuity, as indexed by PAT, predicted accuracy for the novel vowel /y/, which requires the establishment of a new motor pattern constrained by somatosensory target regions.

In contrast, production variability may index the stability of sensorimotor mappings once phonetic categories are established (Kartushina and Frauenfelder, 2014; Guenther, 2016). Within this framework, learners who have developed more stable feedforward motor programs for a given L2 sound are expected to produce it more consistently and accurately. This interpretation is consistent with our finding that lower variability predicted higher accuracy for /u/, a vowel for which experienced learners show ongoing individual variation in the stability of their feedforward motor programs.

Although the present study does not directly assess neural activity, this interpretation is consistent with prior neuroimaging and neurophysiological work demonstrating that individual differences in speech learning and production are associated with variability in auditory, somatosensory, and motor cortical engagement (Reiterer et al., 2011; Simmonds et al., 2011; Lametti et al., 2012). Future studies combining behavioral and neuroimaging approaches will be necessary to directly examine how these sensorimotor representations support individual differences in L2 speech production accuracy.

### 4.7 Limitations of the current study

There are several limitations to the present study. First, production data were collected from participants with varying levels of L2 learning experience, which may have confounded the effects of some predictors. Future studies should control for L2 learning background by including an equal number of beginner and more experienced learners with comparable experience, to better examine how predictors of L2 production accuracy might differ across proficiency levels.

In addition, our use of isolated vowel stimuli may have contributed to ceiling effects in production accuracy and variability, especially for relatively simple vowel targets like /y/. Additionally, this study focused on only two Mandarin vowels. Future research is needed to explore whether the current findings generalize to the learning of other L2 vowels, particularly those that are either more perceptually confusable or phonetically novel across different languages.

## 5 Conclusion

The present study investigated predictors of production accuracy for Mandarin vowels in English late learners of Mandarin. Among the four examined predictors—auditory acuity, somatosensory acuity, production variability, phonological awareness—production variability was found to be the only significant predictor for Mandarin /u/, a perceptually similar L2 vowel to English speakers. In contrast, somatosensory acuity, as measured by PAT, significantly predicted production accuracy for Mandarin /y/, a perceptually new L2 vowel. These findings suggest that the success of L2 speech learning is influenced by the interplay between learners’ sensory profiles and the degree of phonetic similarities between L1 and L2 sounds.

## Data Availability

The datasets presented in this study can be found in online repositories. The names of the repository/repositories and accession number(s) can be found at: https://osf.io/9qt5j/.

## Footnotes

1
www.gorilla.sc
